# Prenatal Diagnosis of Immune-Mediated Fetal Complete Heart Block Revealing Maternal Systemic Lupus Erythematosus

**DOI:** 10.7759/cureus.104433

**Published:** 2026-02-28

**Authors:** Rim Laaboudi, Hounaida Mahfoud, Safae Benjelloun, Fatima Elhassouni

**Affiliations:** 1 Obstetrical Gynecology and High-Risk Pregnancy, Maternity Souissi, Ibn Sina University Hospital, Rabat, MAR; 2 Obstetrics and Gynecology, Ibn Sina University Hospital, Rabat, MAR; 3 Obstetrics and Gynecology, The Faculty of Medicine and Pharmacy of Rabat, Rabat, MAR; 4 Oncology and High-Risk Pregnancies, Maternity Souissi, Ibn Sina University Hospital, Rabat, MAR

**Keywords:** anti-ssa/ro antibodies, congenital heart block, fetal echocardiography, hydroxychloroquine, systemic lupus erythematosus

## Abstract

Immune-mediated congenital heart block (CHB) is a rare but severe manifestation of neonatal lupus, resulting from transplacental transfer of maternal anti-SSA/Ro and anti-SSB/La antibodies. It is associated with significant fetal and neonatal morbidity and mortality and may reveal previously undiagnosed maternal autoimmune disease. We report the case of a 34-year-old multigravida woman with no known systemic disease, referred at 29 weeks of gestation for suspected fetal arrhythmia. Fetal echocardiography revealed complete atrioventricular block associated with myocardial hypertrophy and pericardial effusion. Maternal immunologic screening demonstrated high titers of anti-SSA/Ro and anti-SSB/La antibodies, leading to the diagnosis of systemic lupus erythematosus. Despite multidisciplinary management and antenatal planning, the neonate developed severe postnatal bradyarrhythmia and heart failure, resulting in death on day 4 of life. This case highlights the pathogenic mechanisms, diagnostic challenges, and therapeutic strategies of immune-mediated CHB. Early detection through serial fetal echocardiography and maternal antibody screening is essential, as established complete CHB remains largely irreversible. Preventive therapy with hydroxychloroquine has emerged as the most effective strategy to reduce recurrence risk by more than 50% in subsequent pregnancies, based on recent prospective trials. Immune-mediated CHB may be the first manifestation of maternal autoimmune disease. Systematic immunologic screening, close fetal surveillance, and preventive treatment with hydroxychloroquine are crucial to optimize maternal and neonatal outcomes.

## Introduction

Immune-mediated congenital heart block (CHB) is a rare but serious manifestation of neonatal lupus, caused by the transplacental transfer of maternal anti-SSA/Ro and anti-SSB/La antibodies [1,2]. These autoantibodies trigger complement-mediated inflammation and fibrosis within the fetal atrioventricular (AV) conduction system, leading to progressive bradycardia that may evolve into complete and irreversible heart block if not detected early [3].

Although rare, with an estimated incidence of 1 in 15,000 to 20,000 live births, CHB is associated with high perinatal morbidity and mortality [4]. Among women positive for anti-SSA/Ro or anti-SSB/La antibodies, the risk is approximately 2% in the first affected pregnancy and increases to 18-20% in subsequent pregnancies [5,6].

CHB typically develops between 16 and 24 weeks of gestation, coinciding with the maturation of the fetal conduction system and active maternal IgG transfer [7]. While most cases occur in mothers with known autoantibodies, CHB may also reveal previously unrecognized autoimmune disease, underscoring the need for systematic maternal antibody screening and serial fetal echocardiography [8].

Advances in prenatal diagnosis and preventive therapy, particularly with hydroxychloroquine (HCQ), have significantly improved outcomes [9]. The landmark PATCH (Preventive Approach to Congenital Heart Block with Hydroxychloroquine) trial demonstrated that HCQ initiated before 10 weeks of gestation reduces the recurrence risk of cardiac neonatal lupus by more than 50%, from a historical rate of 18% to 7.4% [9]. However, despite these advances, perinatal morbidity and mortality remain substantial in established cases, highlighting the critical importance of early detection, multidisciplinary management, and comprehensive maternal counseling to optimize both fetal and maternal prognosis [4,9].

## Case presentation

A 34-year-old, gravida 4, para 3 woman, with no known systemic illness, was referred to our department at 29 weeks' gestation for suspected fetal arrhythmia. She reported chronic joint pain but had never undergone rheumatologic evaluation. Her obstetric history included one unexplained neonatal death.

**Figure 1:**
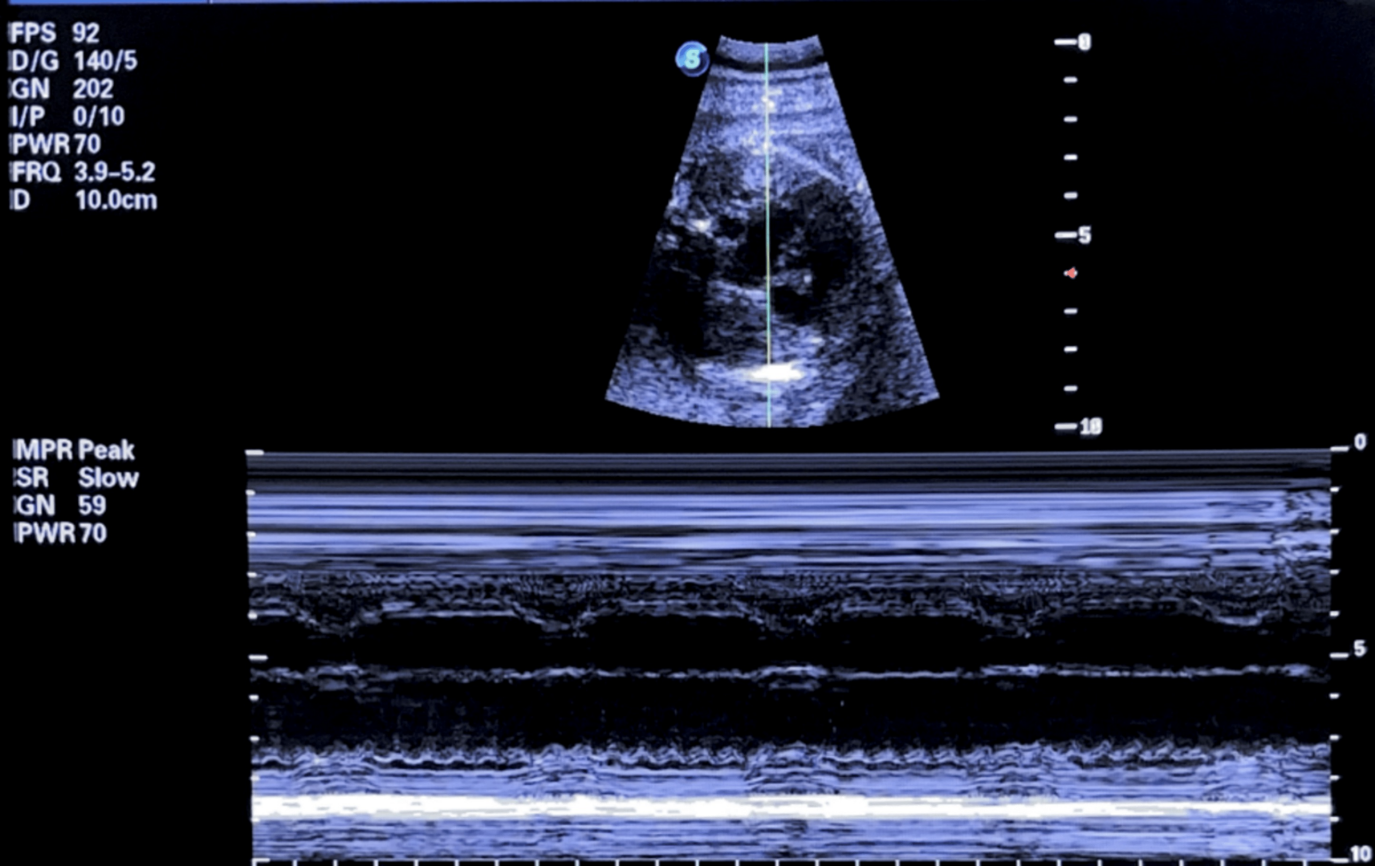
Fetal echocardiography in M-mode showing complete atrioventricular block Fetal M-mode echocardiography at 29 weeks' gestation showing complete atrioventricular block with a ventricular rate of 59 bpm and complete AV dissociation.

Ultrasound revealed a breech fetus with biometric parameters consistent with gestational age. Fetal echocardiography demonstrated myocardial hypertrophy, moderate pericardial effusion, reduced contractility, and AV dissociation suggestive of complete (third-degree) AV block with a ventricular rate of 55 bpm (Figure 1).

**Figure 2:**
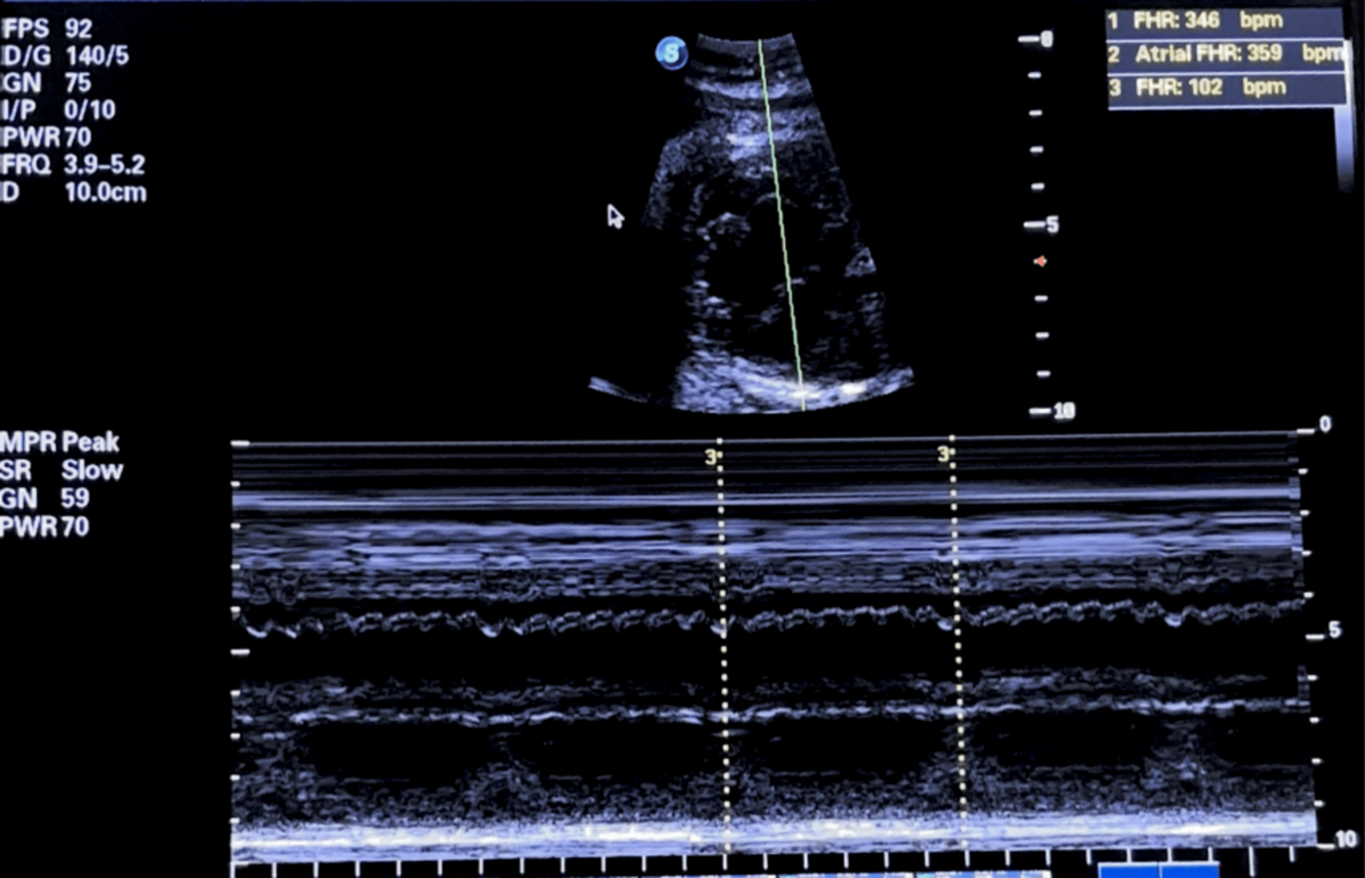
Fetal M-mode showing severe atrioventricular dissociation with bradycardia M-mode echocardiography shows complete AV dissociation: atrial rate: 359 bpm, ventricular rate: 102 bpm (ratio 3:1), confirming third-degree AV block with complete absence of AV conduction.

M-mode echocardiography confirmed complete AV dissociation with independent atrial and ventricular mechanical activity (Figure 2).

Lupus was suspected in view of this complete AV block, and autoimmune screening showed high titers of anti-SSA/Ro (100 IU/mL), anti-SSB/La (71 IU/mL), and anti-dsDNA (28 IU/mL), confirming the diagnosis of systemic lupus erythematosus.

Following confirmation of maternal lupus, treatment combining HCQ (400 mg daily) and corticosteroids (prednisone 20 mg daily) was initiated, resulting in marked clinical improvement in the patient's joint symptoms and general well-being.

At 34 weeks of gestation, the patient presented with premature rupture of membranes associated with fetal distress (baseline FHR 55 bpm). An emergency cesarean section resulted in the delivery of a male neonate weighing 2,600 g, with Apgar scores of 9-10-10. Given the antenatally diagnosed severe bradyarrhythmia, the newborn had been scheduled for immediate postnatal pacemaker implantation in collaboration with the cardiopediatric team. However, his clinical status deteriorated precipitously after birth, with the onset of profound bradyarrhythmia and heart failure refractory to high-dose catecholamines and mechanical ventilation. Pacemaker implantation could not be undertaken, and the infant died on day 4 of life due to irreversible cardiorespiratory failure.

## Discussion

Immune-mediated CHB is a severe manifestation of neonatal lupus resulting from the transplacental passage of maternal IgG autoantibodies, primarily anti-SSA/Ro and anti-SSB/La. These antibodies target fetal cardiomyocytes and components of the cardiac conduction system, initiating complement-mediated inflammation, fibrosis, and calcification within the AV node and surrounding tissues [3]. This autoimmune injury leads to progressive conduction delay that may culminate in irreversible third-degree AV block if not detected early.

At the molecular level, maternal anti-Ro/SSA and anti-La/SSB antibodies bind to their cognate antigens expressed on apoptotic fetal cardiomyocytes, forming immune complexes that inhibit the physiological clearance of apoptotic cells [10]. This leads to the accumulation of cellular debris, triggering macrophage infiltration, complement activation, and sustained inflammation that ultimately results in fibrosis and calcification of the AV node. Recent evidence has identified type I interferon as a key mediator in this cascade, with elevated maternal SIGLEC-1 expression serving as a potential biomarker for disease risk [10].

The onset of CHB typically occurs between 16 and 24 weeks of gestation, corresponding to the period of fetal cardiac conduction system maturation and active transplacental transfer of maternal IgG [7]. Earlier lesions may be subclinical, manifesting only as PR interval prolongation, which underscores the importance of serial fetal cardiac monitoring in seropositive mothers.

Fetal echocardiography remains the cornerstone of prenatal diagnosis and is recommended for all pregnancies in women with anti-SSA/Ro or anti-SSB/La antibodies [11]. M-mode and tissue Doppler imaging enable visualization of mechanical AV dissociation, while assessment of the mechanical PR interval helps identify first- and second-degree blocks before progression to complete CHB [8]. The American Society of Echocardiography guidelines emphasize the critical importance of comprehensive fetal cardiac assessment, including evaluation of cardiac function, rhythm abnormalities, and structural anomalies [11].

Associated findings, such as pericardial effusion, myocardial hypertrophy, tricuspid regurgitation, and umbilical artery Doppler abnormalities, indicate advanced myocardial involvement and poor hemodynamic adaptation [8]. These sonographic markers, when recognized early, can guide therapeutic interventions and close monitoring to prevent fetal demise.

When fetal AV block is identified, a comprehensive differential diagnosis is essential to distinguish immune-mediated CHB from other etiologies with distinct prognostic and therapeutic implications. Non-immune congenital AV block typically occurs in structurally normal hearts in the absence of maternal anti-SSA/Ro or anti-SSB/La antibodies and generally carries a more favorable prognosis [12].

Once complete CHB is established, it is largely irreversible due to extensive fibrosis and scarring of the conduction tissue [13]. Current therapeutic approaches focus on managing complications rather than reversing the block itself. Transplacental treatment with fluorinated corticosteroids (dexamethasone or betamethasone) aims to reduce myocardial inflammation and has demonstrated efficacy in cases of incomplete block or evolving cardiomyopathy, but remains controversial for established complete CHB [14,15].

Intravenous immunoglobulin (IVIG) has emerged as a promising therapeutic option, particularly for incomplete heart block and evolving conduction abnormalities detected before progression to complete CHB [10]. Recent case series have demonstrated that IVIG administered at doses of 1 g/kg for two days can result in reversion to normal sinus rhythm in fetuses and neonates with prolonged PR intervals. In a 10-year retrospective study, all five neonates with first-degree heart block treated with IVIG reverted to normal sinus rhythm, providing evidence for the effectiveness of early immunomodulatory intervention. The proposed mechanism involves modulation of Fc receptor-mediated inflammatory responses, neutralization of pathogenic autoantibodies, and reduction of complement-mediated tissue injury. For more advanced cases, combination therapy with IVIG (1 g/kg every two weeks), dexamethasone, and plasmapheresis has shown promise, with reported reversion to normal sinus rhythm in second-degree blocks and hemodynamic stabilization in third-degree blocks. While further prospective trials are needed to establish optimal protocols, IVIG represents a valuable addition to the therapeutic armamentarium for managing incomplete conduction disease and preventing progression to irreversible complete heart block [10].

The landmark PATCH trial provided robust evidence that HCQ initiated before 10 weeks of gestation reduces recurrence risk by more than 50%, from 18% to 7.4%. This breakthrough has established HCQ as the standard of care for anti-Ro-positive mothers with previous affected pregnancies [9]. The protective mechanism is thought to involve modulation of toll-like receptor signaling, reduction in inflammatory cytokine production, and decreased autoantibody-mediated tissue injury [9].

Maternal management in this case exemplifies several critical principles. First, the diagnosis of systemic lupus erythematosus was prompted solely by the prenatal finding of fetal CHB, highlighting that immune-mediated cardiac conduction disease may be the sentinel event revealing previously subclinical or undiagnosed maternal autoimmune disease. Systematic screening for anti-SSA/Ro and anti-SSB/La antibodies is therefore essential in any case of unexplained fetal bradycardia or AV block [8,11].

Long-term outcomes in children with immune-mediated CHB vary considerably. Those requiring permanent pacing often develop normally, though they remain at increased risk for complications, including endocardial fibroelastosis, dilated cardiomyopathy, and sudden cardiac death [16]. Approximately 50-70% of affected neonates require pacemaker implantation, typically within the first weeks to months of life [4,5]. However, survival rates have improved substantially over recent decades, with contemporary cohorts reporting one-year survival rates exceeding 90% when optimal multidisciplinary care is available [5,13].

The present case underscores the importance of early prenatal detection and meticulous multidisciplinary planning. Despite comprehensive antenatal preparation, the neonate's clinical course deteriorated rapidly postnatally, precluding pacemaker implantation. This tragic outcome illustrates that even with optimal care, some cases of immune-mediated CHB result in irreversible myocardial dysfunction that cannot be adequately managed with pacing alone.

Future research directions should focus on developing more sensitive biomarkers for early detection of myocardial injury, exploring novel therapeutic approaches to reverse established CHB, and establishing standardized protocols for long-term cardiac surveillance in children born to anti-Ro-positive mothers.

## Conclusions

Prenatal recognition of immune-mediated fetal CHB represents a pivotal opportunity for both fetal and maternal care, as it may reveal previously undiagnosed maternal autoimmune diseases such as systemic lupus erythematosus. Systematic screening for anti-SSA/Ro and anti-SSB/La antibodies in cases of unexplained fetal bradycardia, combined with serial fetal echocardiographic monitoring between 16 and 26 weeks of gestation, enables early detection of conduction abnormalities and timely intervention. The integration of HCQ as preventive therapy, based on robust evidence from the PATCH trial, has significantly improved outcomes by reducing recurrence rates in subsequent pregnancies by more than 50%.

A multidisciplinary approach involving obstetricians, fetal cardiologists, rheumatologists, and neonatologists is essential for optimal maternal and fetal outcomes. However, once complete heart block is established, it remains largely irreversible, emphasizing the critical importance of prevention over treatment. Future research should focus on developing more sensitive biomarkers for early myocardial injury detection and novel therapeutic approaches to improve long-term outcomes for affected neonates and their mothers.
